# *Acanthamoeba* as a microbial Trojan horse: intracellular endosymbionts, adaptive immune evasion and antimicrobial resistance dissemination from a one health perspective

**DOI:** 10.3389/fimmu.2026.1874010

**Published:** 2026-09-22

**Authors:** Gen Yan, Yinghua Xuan

**Affiliations:** 1 Department of Radiology, The Second Affiliated Hospital of Xiamen Medical College, Xiamen, Fujian, China; 2 Department of Basic Medical Science, Xiamen Medical College, Xiamen, China

**Keywords:** *Acanthamoeba*, adaptive immunity, antimicrobial resistance, free-living amoebae, horizontal gene transfer, one health, Trojan horse

## Abstract

Antimicrobial resistance (AMR) is a global public health crisis driven not only by clinical antibiotic misuse but also by environmental reservoirs that remain insufficiently characterized. Free-living amoebae of the genus *Acanthamoeba* are ubiquitous eukaryotic protists that graze on diverse bacterial communities via phagocytosis, yet certain clinically important pathogens—including *Legionella pneumophila*, *Mycobacterium* spp. and *Pseudomonas aeruginosa*—have evolved sophisticated mechanisms to survive and replicate within the amoebal phagosome. This intracellular niche serves simultaneously as an evolutionary training ground for bacterial virulence and immune evasion, a physical shelter against environmental biocides, and a hotspot for horizontal gene transfer (HGT) of resistance determinants. Strikingly, the molecular strategies used to subvert amoebal killing are mechanistically conserved with those deployed against human macrophages, effectively shielding amoeba-trained bacteria from both innate and adaptive immune clearance. Recent studies demonstrate that *Acanthamoeba*-associated bacteria exhibit significantly elevated multidrug resistance indices compared with free-living counterparts, that plasmid-borne carbapenem-resistance genes can transfer between co-ingested species within the phagosome, and that the highly resistant cyst stage shields intracellular bacteria from chlorine disinfection at concentrations exceeding water-treatment standards. This review provides a comprehensive synthesis of the molecular and immunological mechanisms underlying intracellular bacterial survival within *Acanthamoeba*, evaluates the evidence for AMR amplification through protist–bacteria interactions, and frames these processes within the One Health paradigm connecting environmental reservoirs to clinical and veterinary outcomes.

## 1 Introduction

Antimicrobial resistance (AMR) has been recognized by the World Health Organization as one of the top ten global public health threats. Current projections estimate that AMR-attributable deaths may reach 10 million annually by 2050 if left unchecked (1). While the conventional paradigm of AMR focuses primarily on clinical settings, a growing body of evidence has revealed that environmental reservoirs play a critical and underestimated role in the maintenance, amplification and dissemination of resistance genes across ecological boundaries (2).

Among these environmental reservoirs, free-living amoebae (FLA) of the genus *Acanthamoeba* occupy a uniquely significant ecological niche. *Acanthamoeba* species are ubiquitous protists found in virtually all environments—soil, freshwater, seawater, swimming pools, cooling towers, hospital water systems, contact lens solutions and even the human nasal mucosa (3, 4). Their life cycle alternates between a metabolically active trophozoite stage, during which they graze on microbial communities via phagocytosis, and a highly resistant cyst stage that can withstand desiccation, UV radiation, temperature extremes and chemical disinfection (5).

The concept of *Acanthamoeba* as a ‘microbial Trojan horse’ was proposed based on the observation that certain bacteria, rather than being digested following phagocytosis, have evolved mechanisms to survive and even replicate within the intracellular milieu (6, 7). This paradigm encompasses a diverse array of clinically important pathogens, including *L. pneumophila*, *Mycobacterium* spp., *P. aeruginosa*, *Vibrio cholerae* and *Francisella tularensis*, as well as giant viruses such as *Mimivirus* and Pandoravirus (8, 9). The evolutionary arms race between *Acanthamoeba* and its intracellular prey has profound implications for human health, as the molecular machinery employed by bacteria to resist amoebal digestion is remarkably conserved with that required for survival within human macrophages and, critically, for evasion of adaptive immune surveillance (10, 11).

The emergence of AMR has prompted renewed interest in these interactions. Recent studies have demonstrated that bacteria associated with *Acanthamoeba* exhibit significantly higher levels of multidrug resistance compared with free-living bacteria in the same environment (12). Furthermore, the phagosomal compartment has been shown to serve as a unique ‘melting pot’ that facilitates HGT between co-ingested species, including the transfer of plasmid-borne resistance determinants (13, 14). These findings position *Acanthamoeba* not merely as a passive refuge for resistant organisms, but as an active contributor to AMR evolution and to the generation of pathogens pre-adapted to evade host immunity.

This review provides a comprehensive synthesis of the current understanding of *Acanthamoeba* as a microbial Trojan horse within the One Health framework. We examine the molecular mechanisms governing intracellular survival, the intersection of amoebal training with adaptive immune evasion, the evidence for AMR amplification through protist–bacteria interactions, and the ecological and clinical implications of these processes.

## 2 Biology of *Acanthamoeba*: the environmental predator and inadvertent host

### 2.1 Taxonomy, morphology and life cycle

*Acanthamoeba* belongs to the supergroup Amoebozoa and is classified into more than 20 genotypes (T1–T22) based on 18S ribosomal RNA gene sequencing (15). The T4 genotype is the most prevalent globally and is most frequently associated with human infections, including *Acanthamoeba* keratitis (AK) and granulomatous amoebic encephalitis (GAE) (16, 17). The life cycle encompasses two distinct stages: the trophozoite (15–45 μm), an irregularly shaped cell characterized by acanthopodia that mediate locomotion and adhesion, and the cyst (10–25 μm), a double-walled dormant form conferring extraordinary environmental resistance (18).

A landmark multi-omics study by Bernard et al. (19) mapped the transcriptomic, proteomic and phosphoproteomic changes during early encystment of *Acanthamoeba castellanii*, identifying over 150,000 previously undescribed transcripts and revealing rapid phospho-regulation of cytoskeletal and translational machinery within the first hour of encystment induction. This molecular atlas has provided critical insights into the protective mechanisms that render cysts resistant to biocides and enable the sequestration of intracellular bacteria within a virtually impenetrable biological shell.

### 2.2 Phagocytosis and the intracellular environment

*Acanthamoeba* trophozoites are professional phagocytes that share remarkable ultrastructural and functional similarities with mammalian macrophages. Both cell types employ receptor-mediated phagocytosis, generate reactive oxygen species (ROS) and utilize phagosome–lysosome fusion as the primary microbicidal mechanism (11, 20). Following internalization via the mannose-binding protein, bacteria are enclosed in phagosomes that undergo acidification and fusion with lysosomes, resulting in enzymatic degradation. The two divergent fates of phagocytosed bacteria—normal killing versus intracellular persistence—are illustrated in **Figure 1**.

**Figure 1:**
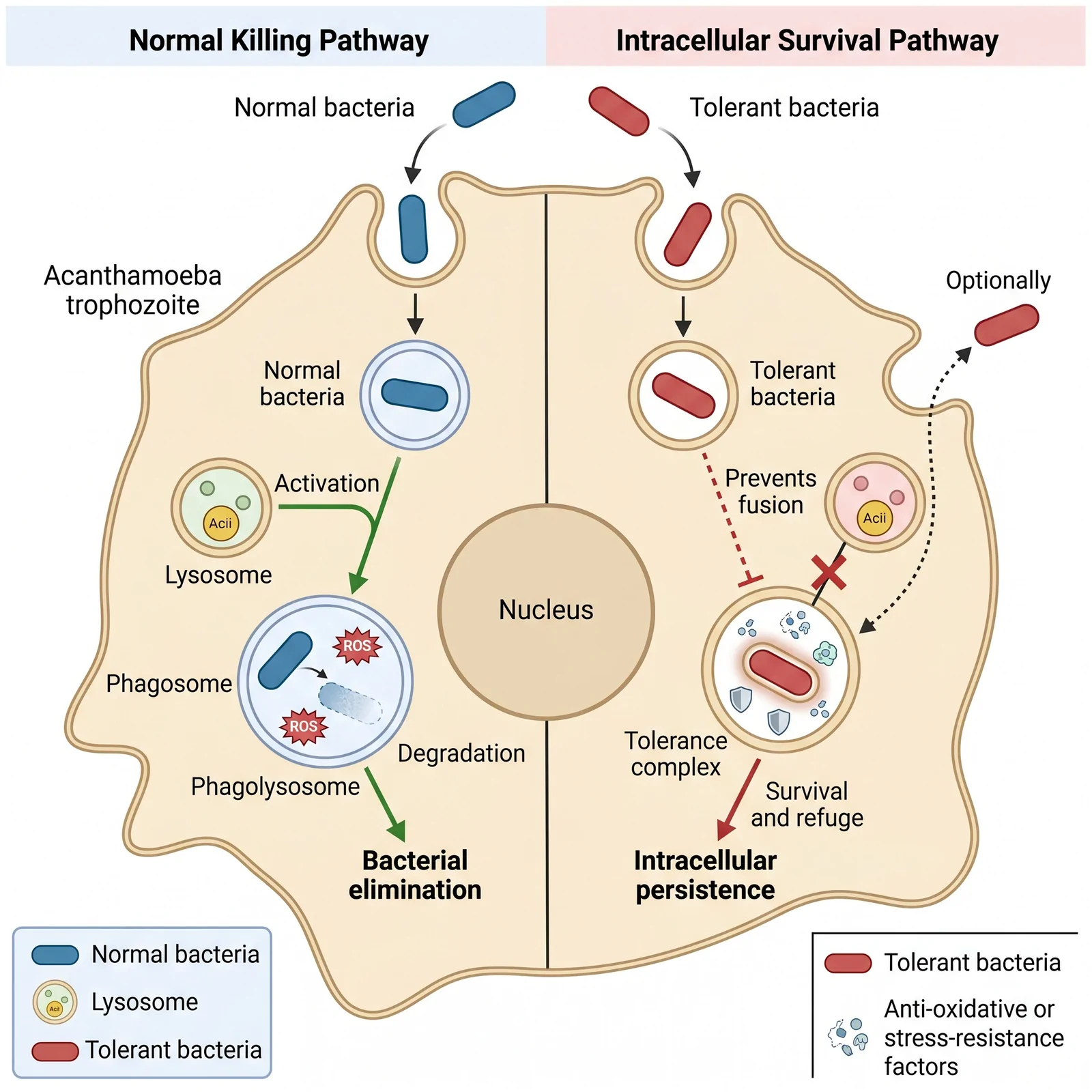
Two divergent fates of bacteria within the *Acanthamoeba* trophozoite. Schematic illustration of an *Acanthamoeba* trophozoite depicting two contrasting intracellular pathways following bacterial phagocytosis. Left panel (normal killing pathway): susceptible bacteria (blue) are engulfed into a phagosome. Lysosomal activation leads to phagosome–lysosome fusion, forming a phagolysosome where reactive oxygen species (ROS) and acid hydrolases degrade the bacteria, resulting in bacterial elimination. Right panel (intracellular survival pathway): tolerant bacteria (red), including species such as *Legionella pneumophila*, *Mycobacterium* spp. and *Pseudomonas aeruginosa*, deploy effector proteins and stress-resistance factors to prevent phagosome–lysosome fusion and inhibit ROS production. These bacteria establish a modified tolerance complex (e.g. *Legionella*-containing vacuole), achieving intracellular persistence. Some bacteria may optionally escape the host cell to re-infect neighboring amoebae. ROS, reactive oxygen species.

However, bacteria that evolve strategies to survive within *Acanthamoeba* phagosomes can deploy identical mechanisms to evade killing by human immune cells. This ‘training ground’ hypothesis is supported by compelling evidence: *Mycobacterium avium* cultured within *Acanthamoeba* demonstrates enhanced virulence and increased antibiotic resistance compared with axenically grown bacteria (reviewed in 21), and *P. aeruginosa* strains recovered from within *Acanthamoeba* exhibit reduced virulence phenotypes—including diminished motility, pyoverdine and rhamnolipid production, biofilm formation and killing of *Caenorhabditis elegans*—demonstrating that amoebal passage can select for attenuated as well as enhanced virulence depending on the species and trait examined (22). Importantly, and because lysis of the amoebal host can itself be a programmed outcome of infection (23), upon release from lysed amoebae, such pre-conditioned bacteria encounter not only innate immune cells but also the adaptive immune system—and the traits refined in amoebal phagosomes afford a substantial competitive advantage in this encounter, as elaborated in Section 4.

## 3 Molecular mechanisms of intracellular bacterial survival

The molecular strategies employed by amoeba-resisting bacteria to subvert *Acanthamoeba* killing are diverse but share common themes of phagosome manipulation, antioxidant defense and immune evasion. Three major pathogens—*L. pneumophila*, *Mycobacterium* spp. and *P. aeruginosa*—exemplify distinct yet convergent approaches to intracellular survival, as summarized in **Figure 2**.

**Figure 2:**
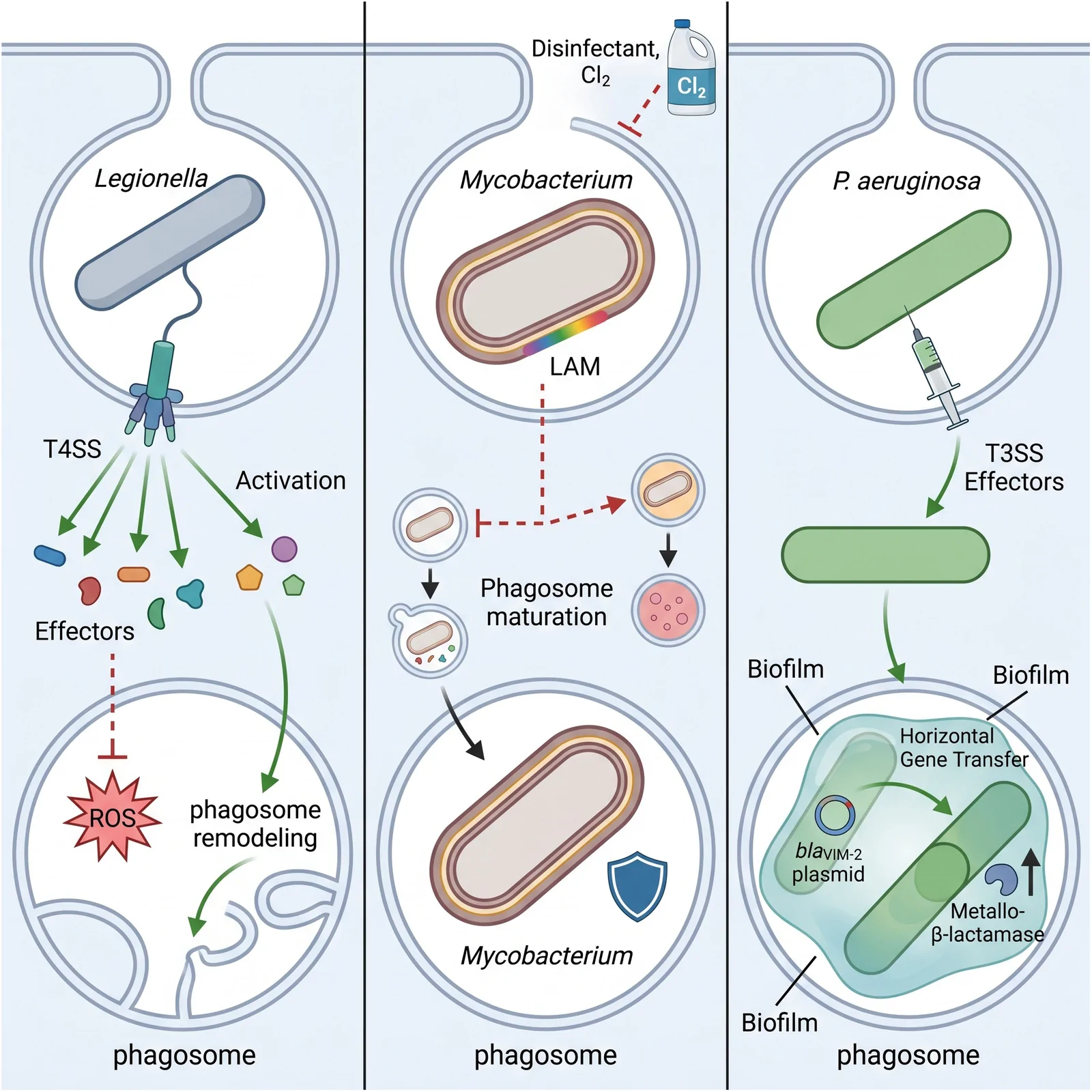
Pathogen-specific molecular mechanisms of intracellular survival within *Acanthamoeba*. Three-panel schematic comparing the distinct survival strategies employed by major amoeba-resisting bacterial pathogens within the *Acanthamoeba* phagosome. **(A)** *Legionella pneumophila*: The Icm/Dot type IV secretion system (T4SS) injects over 300 effector proteins into the host cytoplasm, which inhibit ROS production, block phagosome acidification, and recruit endoplasmic reticulum-derived membranes to remodel the phagosome into a permissive *Legionella*-containing vacuole (LCV). **(B)** *Mycobacterium* spp.: The lipid-rich cell wall, particularly lipoarabinomannan (LAM), arrests phagosome maturation by blocking the Rab5–Rab7 transition and inhibiting phagosome–lysosome fusion. Mycobacteria persist within the arrested phagosome and can survive within amoebal cysts, resisting chlorine disinfection (Cl_2_) at concentrations exceeding standard water treatment protocols. **(C)** *Pseudomonas aeruginosa*: The type III secretion system (T3SS) delivers effector proteins (ExoS, ExoT, ExoU, ExoY) that disrupt host cytoskeletal dynamics. Intracellular bacteria form biofilm-like aggregates and the phagosomal environment facilitates horizontal gene transfer (HGT), including transfer of *bla_VIM-2_*-encoding plasmids conferring metallo-β-lactamase activity and carbapenem resistance. T4SS, type IV secretion system; T3SS, type III secretion system; ROS, reactive oxygen species; LAM, lipoarabinomannan; HGT, horizontal gene transfer.

### 3.1 *Legionella pneumophila*: paradigm of amoebal exploitation

*L. pneumophila* represents the most extensively studied model of intracellular survival within *Acanthamoeba*. The bacterium employs its Icm/Dot type IV secretion system (T4SS) to translocate over 300 effector proteins into the host cell, subverting multiple cellular processes to create a permissive replicative niche termed the *Legionella*-containing vacuole (LCV) (24, 25). These effectors prevent phagosome acidification via SidK-mediated inhibition of V-ATPase, block phagosome–lysosome fusion through SdhA and LegC7 and recruit endoplasmic reticulum-derived membranes to the LCV surface via DrrA, LidA and SidC.

Hay et al. (26) demonstrated that *L. pneumophila* actively modulates the antioxidant defenses of *A. castellanii* during infection. Transcriptomic analysis revealed upregulation of host antioxidant defense transcripts at 6 h post-infection, leading to decreased ROS quantities at 24 h. Metabolomic profiling identified 97 intracellular metabolites disrupted in a T4SS-dependent manner, with four metabolites potentially contributing to the maintenance of a low-oxidative-stress environment favorable for bacterial replication.

Chromosome-scale genome assemblies of *A. castellanii* (27) have further revealed that *Legionella* infection induces significant chromatin reorganization in the host, suggesting epigenetic-level manipulation of gene expression programs. The conservation of the Icm/Dot T4SS and many effector proteins between amoebal and macrophage infection underscores the direct link between environmental protist–bacteria interactions and human disease pathogenesis (28).

### 3.2 Mycobacteria: persistent intracellular residents

Non-tuberculous mycobacteria (NTM), including *Mycobacterium avium* complex, *M. abscessus* and *M. marinum*, represent another major group of amoeba-resisting bacteria. Studies have demonstrated that all tested *Mycobacterium* species (26 out of 26) can survive within *Acanthamoeba* trophozoites for at least 5 days and persist within cysts for up to 15 days (29). Intracellular mycobacteria within cysts survived exposure to free chlorine at 15 mg l^-^¹ for 24 h, far exceeding standard water-treatment protocols.

Mycobacterial survival depends on the lipid-rich cell wall, particularly lipoarabinomannan (LAM), which arrests phagosome maturation by blocking the Rab5–Rab7 transition. The ESX-1 type VII secretion system can damage phagosome membrane integrity, facilitating partial escape to the host cytoplasm. Transposon sequencing (Tn-Seq) applied to *M. marinum* infections identified MCE family transporters as essential for intracellular fitness, enabling acquisition of host-derived cholesterol and fatty acids (30).

Clinical isolates of *M. abscessus* from chronic pulmonary infections demonstrate enhanced cytotoxicity toward *Acanthamoeba* compared with early-infection isolates, suggesting progressive selection for traits that enhance both amoebal and macrophage exploitation (31). Mycobacteria cultured within *Acanthamoeba* exhibit increased resistance to rifabutin, azithromycin and clarithromycin, demonstrating that intracellular passage selectively enriches resistant subpopulations (reviewed in 21).

### 3.3 *Pseudomonas aeruginosa* and horizontal gene transfer

*P. aeruginosa* utilizes *Acanthamoeba* as a protective reservoir in clinical environments. Mooney et al. (32) demonstrated that *Acanthamoeba* isolated from hospital water systems harbored viable *P. aeruginosa*, effectively shielding the bacteria from chlorine-based disinfection. The bacterium employs its type III secretion system (T3SS) to inject effector proteins (ExoS, ExoT, ExoU, ExoY) that disrupt host cytoskeletal dynamics and can form biofilm-like intracellular aggregates.

Critically, the phagosome of *Acanthamoeba* serves as a hotspot for HGT among co-ingested bacteria. Sarink et al. (13) provided direct experimental evidence that *A. castellanii* facilitates plasmid transfer between environmental *Pseudomonas* species. Using confocal microscopy, they demonstrated transfer of a *bla_VIM-2_*-encoding plasmid from *P. oleovorans* to *P. aeruginosa* during intracellular co-residence. This is particularly alarming because bla_VIM-2_ encodes a metallo-β-lactamase conferring carbapenem resistance. Furthermore, co-cultured *P. putida* within *Acanthamoeba* survived ciprofloxacin concentrations of 20 μg ml^-^¹—forty times the minimum inhibitory concentration for the naïve strain (0.5 μg ml^-^¹)—whereas bacteria without amoebal protection were killed at MIC levels. However, this transfer phenotype was highly strain-dependent: among 19 *P. aeruginosa* recipient strains tested, amoeba-enhanced plasmid transfer reached statistical significance in only one (strain 957), and although *bla_VIM-2_* also transferred to three further strains, it did so at negligible rates that were not increased by the presence of *A. castellanii*—the original authors explicitly caution that intra-phagosomal AMR gene transfer in this system is highly strain-dependent and should not be generalized without qualification.

The mechanisms facilitating intra-phagosomal HGT likely include both conjugation and transformation. Rahman et al. (33) demonstrated that protozoal food vacuoles enhance transformation in *V. cholerae* through SOS-regulated DNA integration, suggesting that the stress of intracellular survival activates genetic exchange machinery. Consistent with this, adaptation to an amoeba host has been shown to drive selection of virulence-associated traits in Vibrio cholerae (34). Comparative genomic analyses of clinical *Acanthamoeba* isolates have revealed extensive horizontal transfer of AMR genes, further supporting the role of the amoebal niche in genetic exchange (35).

### 3.4 Resistance to bacterial pore-forming toxins: a counterintuitive twist

A counterintuitive dimension of the *Acanthamoeba*–bacteria relationship concerns pore-forming toxins (PFTs), classic bacterial virulence factors—produced by both Gram-positive and Gram-negative species such as *Vibrio* spp., *Bacillus cereus* and Serratia marcescens—that lyse mammalian host cells by inserting oligomeric pores into the plasma membrane. Using high-throughput imaging, confocal microscopy and holographic single-cell tracking across a panel of roughly 28 environmental and pathogenic bacterial species, most of which produce pore-forming toxins, Yabrag et al. (36) demonstrated that *A. castellanii* is, in contrast to murine and human macrophages, broadly resistant to PFT-mediated cytotoxicity. In their assays, bacterial species and cell-free supernatants that killed RAW 264.7 and THP-1 macrophages within hours left *A. castellanii* viable under comparable conditions. Western blotting confirmed that PFT components (e.g., the *Bacillus cereus* NheB subunit) were successfully delivered into the amoeba, ruling out a simple failure of toxin delivery. The authors instead attributed the resistance to differences in membrane sterol composition. Cholesterol—required for oligomerization of many PFTs—is replaced by ergosterol and 7-dehydrostigmasterol in the *Acanthamoeba* membrane (**Figure 3**).

**Figure 3:**
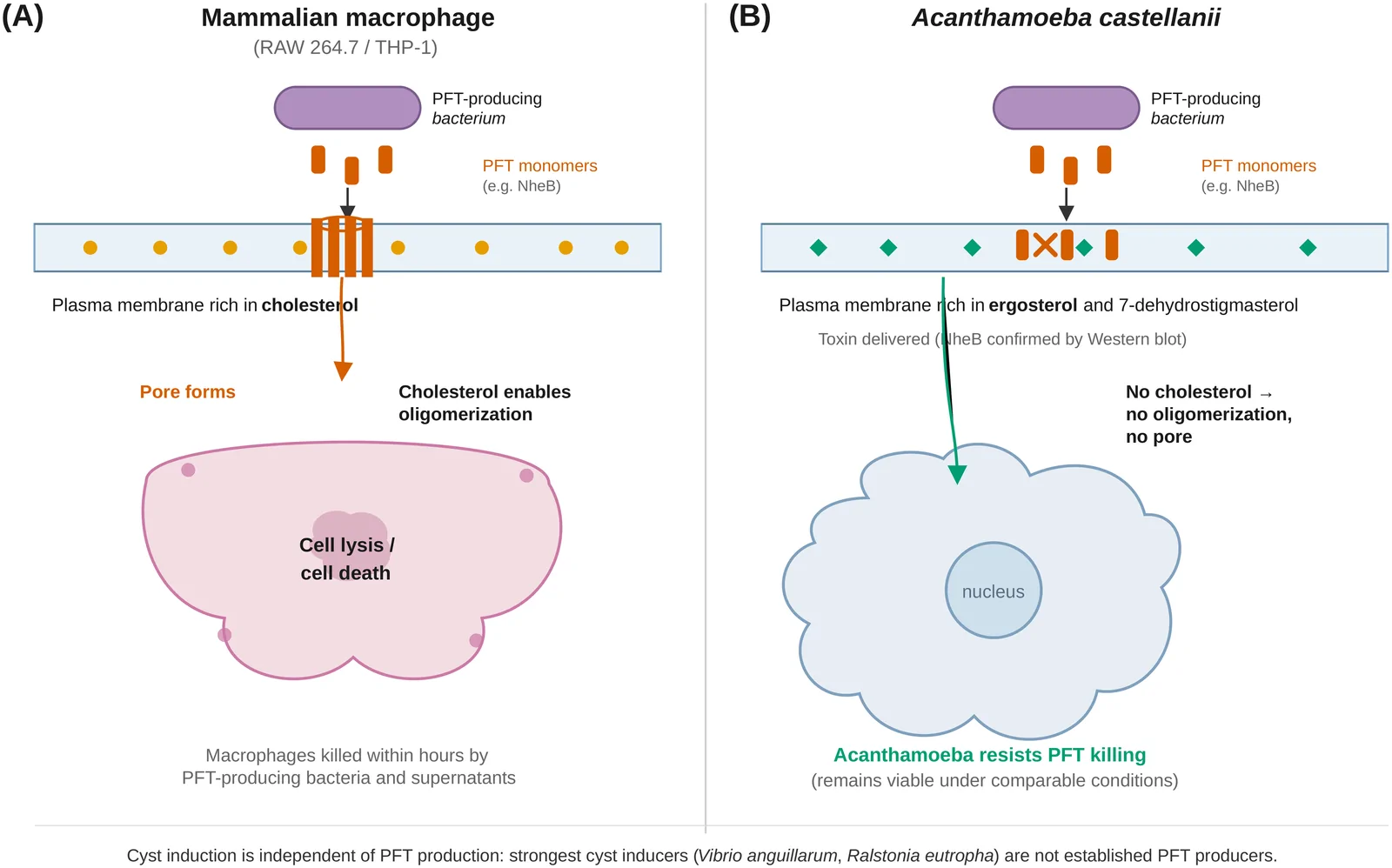
Sterol-dependent resistance of *Acanthamoeba* to bacterial pore-forming toxins (PFTs). **(A)** In mammalian macrophages (e.g. RAW 264.7 and THP-1), PFT monomers secreted by PFT-producing bacteria (for example the *Bacillus cereus* Nhe component, NheB) bind cholesterol in the plasma membrane and oligomerize to form lytic pores, causing membrane permeabilization and cell death within hours. **(B)** In *A. castellanii*, the plasma membrane contains ergosterol and 7-dehydrostigmasterol rather than cholesterol; although PFT delivery still occurs (NheB is detectable in the amoeba by Western blotting), the absence of cholesterol prevents pore oligomerization, so no functional pore forms and the amoeba remains viable under comparable conditions. Cyst induction is independent of PFT production: the strongest inducers of encystment and lysis in this system (*Vibrio anguillarum* and *Ralstonia eutropha*) are not established PFT producers. Based on Yabrag et al. (36).

Notably, cyst induction in *A. castellanii* did not correlate with PFT production: the two bacterial species that most strongly triggered cyst formation and lysis in this study, *Vibrio anguillarum* and *Ralstonia eutropha*, are not established PFT producers, whereas several confirmed PFT-producing species (*P. aeruginosa*, *B. cereus*, *V. cholerae*) induced comparatively little cyst formation. This finding complicates a simple extension of the ‘training ground’ paradigm to PFT-producing pathogens: rather than being killed and thereby selecting for PFT-resistant *Acanthamoeba* lineages, most PFT producers appear to coexist with the amoeba without triggering either defensive encystment or amoebicidal killing. This apparent tolerance may prolong co-residence and provide an additional, toxin-independent route by which these organisms could be shielded from environmental biocides and antibiotics within the phagosome or cyst.

### 3.5 Other clinically important amoeba-resistant bacteria

Beyond *L. pneumophila*, *Mycobacterium* spp. and *P. aeruginosa*, several additional multidrug-resistant nosocomial pathogens are recognized amoeba-resistant bacteria (ARB). *Acinetobacter baumannii* survives and replicates within *A. castellanii*, residing in cytoplasmic vacuoles as trophozoites within 1–3 days of co-culture and persisting between the double cyst walls for several weeks, with amoebal co-culture measurably promoting bacterial growth (37). *Burkholderia cepacia* complex isolates—a major concern for patients with cystic fibrosis—survive, without appreciable replication, within acidified vacuoles of *Acanthamoeba polyphaga*; metabolically active bacteria are required to induce the profuse cytoplasmic vacuolization observed in infected trophozoites (38). *Stenotrophomonas maltophilia*, now ranked among the ten most prevalent multidrug-resistant nosocomial pathogens, goes further and actively replicates within *A. castellanii*: live-cell imaging shows it resides within an acidified, Rab7A-positive *Stenotrophomonas*-containing vacuole that interacts extensively with the endocytic pathway, undergoing multiple rounds of intracellular replication before non-lytic exocytosis (39). Enterobacteriaceae, including *Enterobacter* spp., are also recovered from *Acanthamoeba*-associated biofilms and contribute to the horizontal exchange of AMR genes documented in clinical *Acanthamoeba* isolates (35), although dedicated mechanistic studies of intracellular survival specifically within *Acanthamoeba* remain comparatively scarce for this genus. Collectively, these examples indicate that amoeba resistance is a broadly distributed trait among Gram-negative nosocomial pathogens rather than a peculiarity of the three organisms discussed in Sections 3.1–3.4, reinforcing *Acanthamoeba*’s role as a general-purpose environmental reservoir for multidrug-resistant bacteria (summarized in **Table 1**).

**Table 1:** Major intracellular bacterial pathogens of *Acanthamoeba*: survival mechanisms, associated AMR mechanisms and clinical significance.

Pathogen	Key survival mechanism(s) in *Acanthamoeba*	Associated AMR mechanism(s)	Clinical significance
*Legionella pneumophila*	T4SS effector-mediated phagosome remodeling (SidK, SdhA, LegC7, DrrA/LidA/SidC); ER-derived *Legionella*-containing vacuole (LCV)	Reduced/modulated MHC class II antigen presentation after macrophage infection (Section 4.1)	Legionnaires’ disease; hospital and cooling-tower water-system outbreaks
*Mycobacterium* spp. (*M. avium*, *M. abscessus*, *M. marinum*)	LAM-mediated phagosome-maturation arrest; ESX-1-mediated phagosome damage; survival within cysts	Increased resistance to rifabutin, azithromycin and clarithromycin after amoebal passage (21)	Non-tuberculous mycobacterial pulmonary infections; treatment failures linked to environmental exposure
*Pseudomonas aeruginosa*	T3SS effectors (ExoS, ExoT, ExoU, ExoY); biofilm-like intracellular aggregates	*bla_VIM-2_* plasmid transfer (HGT, strain-dependent, Section 3.3); chlorine and fluoroquinolone tolerance	Hospital water-system reservoir; carbapenem-resistant nosocomial infections
*Acinetobacter baumannii*	Cytoplasmic vacuolar residence as trophozoites (1–3 d); persistence within cysts for several weeks (37)	Widespread multidrug resistance among clinical isolates; amoebal co-culture promotes bacterial growth	Ventilator-associated pneumonia; hospital water and plumbing reservoirs
*Burkholderia cepacia* complex	Non-replicative survival within acidified vacuoles of *A. polyphaga* (38)	Intrinsic multidrug resistance (efflux pumps)	Chronic infection in cystic fibrosis; patient-to-patient and environmental transmission
*Stenotrophomonas maltophilia*	Active intracellular replication within an acidified, Rab7A-positive vacuole; non-lytic exocytosis (39)	Intrinsic multidrug resistance (SmeYZ, SmeDEF efflux pumps)	Emerging top-10 MDR nosocomial pathogen; infections in ventilated/immunocompromised patients
*Enterobacter* spp./Enterobacteriaceae	Recovered from *Acanthamoeba*-associated biofilms; mechanism of intracellular survival less well characterized	AcrAB-TolC efflux-mediated MDR; horizontal transfer of AMR genes documented in *Acanthamoeba* isolates (35)	Healthcare-associated infections; environmental AMR gene reservoir

## 4 Intersection of amoebal training with adaptive immune evasion

The immunological consequences of bacterial passage through *Acanthamoeba* extend well beyond the realm of innate immunity. A central but underappreciated dimension of the Trojan horse paradigm is that the survival toolkit refined within amoebal phagosomes confers bacteria with a formidable capacity to resist adaptive immune clearance upon entry into the human host. Understanding this intersection is essential for framing *Acanthamoeba*-mediated AMR dissemination within an immunological context.

### 4.1 Conservation of immune evasion mechanisms across phagocytic hosts

The remarkable mechanistic conservation between amoebal and macrophage phagosomes has been well established (10, 11). Both compartments impose similar selective pressures: acidification, oxidative burst, lysosomal hydrolases and nutrient restriction. Bacteria that evolve or acquire resistance to amoebal killing—a process that has operated over evolutionary timescales—are therefore pre-adapted to resist macrophage-mediated killing, the first cellular line of innate defense in human tissue (20).

However, the immunological relevance extends beyond macrophage evasion to encompass the subsequent adaptive response. Intracellular bacteria that reside within phagosomes subvert antigen presentation pathways, thereby interfering with the priming of pathogen-specific T cell responses. *L. pneumophila*, for instance, uses its T4SS effectors to inhibit phagosome–lysosome fusion in human macrophages, thereby modulating the processing and major histocompatibility complex (MHC) class II-mediated presentation of bacterial antigens to CD4^+^ T helper cells; notably, dendritic cells—unlike macrophages—retain some capacity to present *Legionella* antigens on MHC class II despite residing in an ER-derived vacuole, indicating that the net effect on CD4^+^ priming is cell-type dependent (40). The identical effector repertoire expressed during amoebal infection suggests that environmental passage through *Acanthamoeba* may directly pre-select for strains with superior antigen presentation evasion capacity in clinical settings.

Analogous mechanisms operate in mycobacteria. LAM-mediated phagosome maturation arrest not only prevents bactericidal fusion in macrophages but also impairs cross-presentation of mycobacterial peptides on MHC class I molecules, has been proposed to blunt the cytotoxic CD8^+^ T lymphocyte (CTL) response critical for controlling intracellular infection, although direct evidence for this cross-presentation defect in amoeba-trained isolates is still lacking. Because passage through *Acanthamoeba* selects for bacteria with enhanced LAM production and phagosome arrest capacity (reviewed in 21), environmental exposure to amoebae may, in effect, enrich for strains that are intrinsically more refractory to T cell-mediated immunity.

### 4.2 Amoebal release and subversion of the adaptive immune response

Upon lytic release from *Acanthamoeba*, amoeba-conditioned bacteria enter the host microenvironment in a state of heightened virulence and resistance that is likely to overwhelm conventional adaptive immune control. Several lines of evidence support this view. First, *M. avium* recovered from intracellular passage through *Acanthamoeba* exhibits increased resistance to killing by human peripheral blood mononuclear cells (PBMCs) ex vivo, suggesting that amoebal training modifies bacterial surface properties in ways that dampen lymphocyte activation (reviewed in 21). Second, amoeba-adapted *P. aeruginosa* isolates show reduced motility, pyoverdine/rhamnolipid production and biofilm formation (22); whether these attenuated-virulence phenotypes also translate into altered LPS/TLR-dependent dendritic cell maturation and T helper cell priming has not been directly tested in this system and remains an open question.

Furthermore, the cyst-mediated release pathway adds an additional layer of immunological complexity. Bacteria liberated from disrupted cysts are shielded within residual cyst wall debris, potentially masking surface epitopes recognized by antibodies and complement. Secretory IgA at mucosal surfaces—the first adaptive barrier encountered by waterborne pathogens—may be similarly impaired in its neutralizing capacity against bacteria presenting modified surface architectures selected during amoebal passage. Consequently, the humoral arm of adaptive immunity, typically effective against extracellular bacteria, may be functionally compromised against amoeba-conditioned pathogens carrying altered antigenic profiles.

### 4.3 Implications for vaccine design and immunotherapy

The intersection of amoebal ecology with adaptive immunity has underexplored but potentially transformative implications for vaccine development and immunotherapy against amoeba-associated pathogens. Conventional vaccine antigens are typically selected from laboratory-grown bacteria and may not represent the antigenic landscape of environmentally conditioned, amoeba-trained strains. If passage through *Acanthamoeba* substantially alters surface antigen expression—as suggested by transcriptomic and proteomic evidence (27)—vaccines designed against axenically cultured bacteria may afford incomplete protection against clinically relevant, amoebal-conditioned isolates.

Conversely, this mechanistic understanding offers novel therapeutic targets. The shared effector systems exploited during amoebal and macrophage infection—particularly the Icm/Dot T4SS and ESX-1 secretion systems—represent candidates for broad-spectrum immunomodulatory strategies. Blocking these secretion systems pharmacologically or eliciting neutralizing antibodies against conserved effector proteins could simultaneously impair both amoebal and macrophage colonization. Moreover, CRISPR–Cas9-based genetic tools now available for *A. castellanii* (41) enable the systematic identification of bacterial genes specifically induced during amoebal infection, providing a rational pipeline for antigen discovery targeting the intracellular, immunoevasive phenotype that amoeba-trained bacteria express upon entering the human host.

## 5 *Acanthamoeba* as an amplifier of antimicrobial resistance

Beyond its role as an immunological training ground, *Acanthamoeba* functions as a highly efficient amplifier of AMR through a convergence of physical, genetic and chemical mechanisms. The aggregate effect of these processes is the generation and release of bacterial communities with resistance profiles substantially elevated above those of their free-living counterparts, with direct implications for clinical infection control and public health.

### 5.1 Enhanced multidrug resistance in amoeba-associated bacteria

Mooney et al. (12), publishing in Environmental Microbiology, provided the most compelling evidence to date for AMR enrichment within *Acanthamoeba*. Investigating *Acanthamoeba*-associated bacterial communities (AAB) and sediment-associated bacterial communities (SAB) in a polluted estuarine system in Navi Mumbai, India, they found that AAB displayed significantly higher multiple antibiotic resistance (MAR) indices, with elevated resistance to aminoglycosides, macrolides, fluoroquinolones and β-lactams. All isolated amoebae belonged to the T4 genotype, suggesting selection for resilient host organisms in contaminated environments.

This enrichment is attributable to at least three non-mutually exclusive mechanisms that operate simultaneously within the amoebal phagosome. First and most directly, the intracellular environment provides physical protection from extracellular antibiotics: the amoebal cell membrane and, during encystment, the double-layered cyst wall create barriers that dramatically reduce intracellular antibiotic concentrations. Sarink et al. (42) demonstrated that *A. castellanii* reduces the efficacy of chlorine-based disinfection against multidrug-resistant *P. aeruginosa* by over 99% compared with free-living bacteria exposed to equivalent concentrations. Second, environmental stresses within the phagosome—oxidative burst, acidification and nutrient deprivation—generate potent selective pressure that disproportionately favors pre-existing resistant subpopulations, effectively purifying resistant variants from initially heterogeneous bacterial communities. Third, the co-localization of phylogenetically diverse bacteria within the same phagosome creates unprecedented opportunities for HGT-mediated resistance acquisition, as discussed in Section 3.3.

A particularly alarming implication of these combined mechanisms concerns last-resort antibiotic classes. The *bla_VIM-2_* carbapenem resistance gene demonstrated to transfer within *Acanthamoeba* phagosomes encodes resistance to carbapenems, the current last line of treatment for multidrug-resistant Gram-negative infections (13). Similarly, the potential for mcr-mediated colistin resistance—the antibiotic of absolute last resort—to undergo intra-phagosomal transfer has not been experimentally excluded and warrants urgent investigation. The convergence of physical protection, selection and genetic exchange within the amoebal niche thus creates conditions uniquely permissive for the evolution of extensively drug-resistant (XDR) and potentially pandrug-resistant (PDR) phenotypes.

### 5.2 Co-selection by potentially toxic elements

An emerging dimension of AMR amplification within *Acanthamoeba* involves co-selection of antibiotic resistance by potentially toxic elements (PTEs) such as heavy metals. Mooney et al. (12) reported significant positive correlations between PTE concentrations (copper, zinc, lead and cadmium) in polluted estuarine sediments and the MAR indices of *Acanthamoeba*-associated bacteria, providing field-scale evidence that metal contamination indirectly promotes antibiotic resistance within the amoebal microbiome.

The mechanistic basis of this co-selection is well established at the molecular level: metal resistance determinants and antibiotic resistance genes are frequently co-located on the same mobile genetic elements, including conjugative plasmids and integrative conjugative elements. Efflux pumps of the resistance-nodulation-division (RND) family, such as MexAB-OprM in *P. aeruginosa* and AcrAB-TolC in Enterobacteriaceae, confer simultaneous tolerance to multiple heavy metals and antibiotics, including fluoroquinolones, β-lactams and aminoglycosides. Consequently, selection pressure exerted by environmental metal contamination—which is widespread in industrialized and peri-urban water bodies receiving agricultural runoff and industrial effluent—may drive enrichment of multi-resistance plasmids within the protected intracellular niche of *Acanthamoeba* without any direct antibiotic exposure.

This co-selection pathway has particular significance in low- and middle-income countries (LMICs), where agricultural antibiotic use, inadequate industrial waste management and heavy-metal-contaminated water bodies frequently co-occur (12). In such settings, the *Acanthamoeba*-associated microbiome may accumulate and amplify resistance determinants through metal-driven co-selection, subsequently exporting AMR-enriched bacteria into municipal water supplies and agricultural produce chains, completing a cycle of environmental–clinical resistance gene flux that operates largely beneath the radar of conventional AMR surveillance programs.

## 6 Cyst-mediated protection: implications for disinfection and persistence

The cyst stage of *Acanthamoeba* represents a formidable barrier to environmental control strategies. The double-walled cyst structure, composed primarily of cellulose and protein, provides resistance to a wide range of biocidal agents (43). Multi-omics analyses have revealed that cyst-wall synthesis involves coordinated reprogramming of sugar metabolism, with glycogen phosphorylase, cellulose synthase and specific lectin families (Jonah, Luke and Leo) playing central roles (19, 44).

Bacteria sequestered within cysts are effectively shielded from antimicrobials. This has been demonstrated for multiple pathogens: *Mycobacterium* spp. survive within cysts under the chlorine-exposure conditions detailed in Section 3.2 (29), and *L. pneumophila* released from cysts exhibits enhanced virulence (24). In healthcare settings, standard disinfection protocols for water distribution systems may fail to eliminate cyst-protected pathogens, creating persistent reservoirs for nosocomial infection (32, 42).

Recent advances in understanding cyst biology, including the phosphoproteomic mapping of early encystment signals (19) and CRISPR–Cas9-amenable genetic manipulation protocols for both *Acanthamoeba* and its giant virus inhabitants (41), offer potential avenues for developing targeted anti-encystation therapies that could simultaneously address *Acanthamoeba* pathogenesis and the reservoir function of cysts for antibiotic-resistant bacteria.

## 7 One health framework: connecting environmental reservoirs to clinical outcomes

The One Health paradigm recognizes that human, animal and environmental health are inextricably linked. **Figure 4** provides a conceptual overview of how *Acanthamoeba* functions as a central hub for AMR dissemination across these interconnected domains, with water distribution systems serving as the primary transmission interface. The stages of this process, from environmental acquisition to clinical, community and veterinary endpoints, are summarized in **Table 2**.

**Figure 4:**
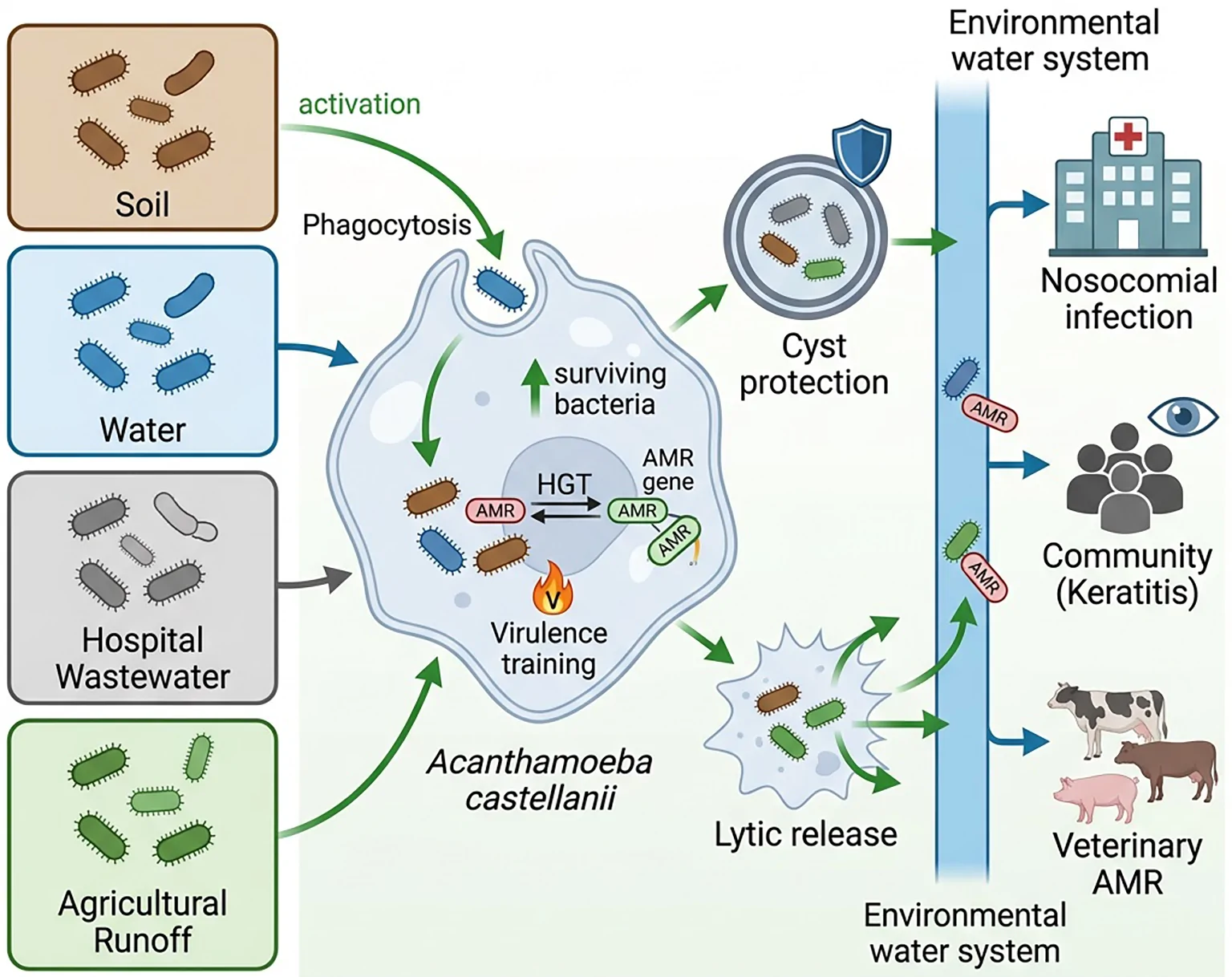
*Acanthamoeba* as a hub for antimicrobial resistance dissemination: a One Health perspective. Conceptual diagram illustrating the central role of *Acanthamoeba castellanii* in the environmental dissemination of antimicrobial resistance (AMR) through interconnected ecological and clinical pathways. Bacteria from diverse environmental sources—soil, natural water, hospital wastewater, and agricultural runoff—are phagocytosed by *Acanthamoeba* trophozoites. Within the intracellular environment, three key processes occur: (i) surviving bacteria undergo horizontal gene transfer (HGT) of AMR genes between co-ingested species; (ii) intracellular passage enhances bacterial virulence and adaptive immune evasion capacity (‘virulence training’); and (iii) bacteria acquire or amplify resistance determinants. Bacteria are subsequently released via two routes: encapsulation within highly resistant cysts (cyst protection pathway) or through host cell lysis (lytic release pathway). Both routes deliver AMR-enriched bacteria into environmental water distribution systems, from which they reach three major endpoints: healthcare facilities (nosocomial infections), communities (contact lens-related *Acanthamoeba* keratitis), and agricultural/veterinary settings (veterinary AMR). AMR, antimicrobial resistance; HGT, horizontal gene transfer; V, virulence.

**Table 2:** Conceptual timeline of *Acanthamoeba*-mediated bacterial training, AMR amplification and transmission, from environmental acquisition to clinical/veterinary endpoints.

Stage	Approximate timescale	Key events	Representative evidence
1. Environmental acquisition	Continuous/ongoing	Free-living bacteria in soil, water and biofilms are phagocytosed by *Acanthamoeba* trophozoites via mannose-binding protein-mediated uptake	Section 2.2 (20)
2. Intracellular persistence and virulence training	Hours to ≥15 days	Amoeba-resisting bacteria block phagosome–lysosome fusion; e.g. *Mycobacterium* spp. survive ≥5 days as trophozoites and persist ≤15 days within cysts; virulence and AMR-evasion traits are selected	Section 3 (21, 29)
3. Horizontal gene transfer	Timescale of intracellular co-residence (days)	Co-ingested bacteria exchange plasmid-borne resistance genes (e.g. *bla_VIM-2_*); transfer efficiency is strain-dependent	Section 3.3 (13)
4. Cyst formation	Initiates within the first hour of induction; wall matures over subsequent hours–days	Coordinated phospho-regulation and cellulose/glycoprotein cyst-wall synthesis produce a biocide-resistant double-walled cyst	Section 2.1, Section 6 (19)
5. Release from the amoebal host	Variable (lytic release or cyst dispersal)	Bacteria are released either by host-cell lysis or by dispersal of intact, biocide-resistant cysts into the environment	Section 6 (24)
6. Transmission to clinical, community and veterinary endpoints	Upon host exposure	AMR-enriched, immune-evasive bacteria reach humans and animals via water systems, causing nosocomial infection, community-acquired keratitis or veterinary disease	Section 7 (32, 45)

### 7.1 Water systems as the critical interface

Water distribution systems represent the primary interface through which *Acanthamoeba*-mediated AMR dissemination impacts human health. *Acanthamoeba* is commonly found in municipal water supplies, hospital plumbing systems, cooling towers and recreational water facilities. Biofilm formation on pipe surfaces creates ecological niches supporting long-term persistence of *Acanthamoeba* and its intracellular bacterial communities. The inadequacy of standard chlorination protocols to eliminate cyst-protected bacteria creates a direct pathway for resistant organisms to reach vulnerable populations, particularly immunocompromised patients in healthcare settings (32).

Beyond hospital plumbing, *Acanthamoeba* and its associated biofilms are also well documented in wastewater treatment plants and municipal drinking water distribution systems, where premise plumbing and storage tanks provide additional niches for long-term persistence. A recent evaluation of disinfection strategies against clinically relevant free-living amoebae isolated from drinking water and plumbing biofilms found that thermal treatment (70 °C) achieved >4 log_10_ reduction of both trophozoites and cysts, outperforming chemical disinfectants including chlorine dioxide and benzalkonium chloride, and concluded that current water-management practices are often insufficient to control biofilm-associated *Acanthamoeba* and its intracellular bacterial passengers (46). These findings underscore the need for dedicated environmental surveillance programmes—incorporating routine *Acanthamoeba* and intracellular-pathogen monitoring alongside conventional indicator-organism testing—across wastewater treatment plants, drinking water utilities and healthcare plumbing networks, rather than relying on chlorine-residual monitoring alone.

The global epidemiology of AK provides indirect evidence for the widespread distribution of these organisms. Aiello et al. (45) estimated approximately 12,953 eyes affected by AK globally in 2023, with a projected 25.5% increase by 2073. Zhang et al. (47) calculated an annual global incidence of 23,561 cases from data across 20 countries. The increasing incidence of AK serves as a sentinel indicator of the broader ecological dynamics of *Acanthamoeba* populations in human-associated water environments.

### 7.2 Clinical and veterinary dimensions

The clinical implications extend beyond direct amoebic infections. Bacteria released from lysed amoebae may enter clinical environments through contaminated water and cause healthcare-associated infections with enhanced resistance profiles. The corresponding increase in resistance to front-line antimycobacterial drugs described in Section 4.2 suggests that environmental exposure to amoebal hosts may contribute to treatment failures in NTM infections. From a veterinary perspective, *Acanthamoeba* has been isolated from various animal hosts and environments associated with livestock production (3). The potential for agricultural environments to serve as reservoirs for *Acanthamoeba*-associated resistant bacteria, particularly in regions with heavy antibiotic use in animal husbandry, remains an important but largely unexplored dimension of the One Health framework.

### 7.3 Global and regional perspectives

The significance of *Acanthamoeba* as an AMR reservoir is likely to vary across geographic and socioeconomic contexts. In LMICs, where water-treatment infrastructure may be inadequate and environmental antibiotic pollution is prevalent, the contribution of protist-mediated AMR dissemination may be disproportionately high (12). Scalable monitoring approaches that integrate protist–bacteria interactions into existing AMR surveillance frameworks are urgently needed, particularly in regions where the AMR burden is greatest. The 2024–2025 outbreak of amoebic meningoencephalitis in Kerala, India—involving *Naegleria fowleri*, *Acanthamoeba* spp. and *Balamuthia mandrillaris*—has been linked to rising water temperatures, inadequate chlorination and coliform contamination, illustrating how climate-driven changes in water quality can amplify free-living amoeba exposure risk in precisely the LMIC settings where AMR surveillance capacity is weakest (48).

## 8 Research gaps and future directions

Despite significant advances in characterizing *Acanthamoeba* as an environmental AMR amplifier and an immunological training ground, critical knowledge gaps remain at multiple levels—from molecular mechanisms to ecological quantification and translational intervention. Addressing these gaps will require coordinated, multidisciplinary efforts spanning environmental microbiology, clinical immunology, genomics and public health.

First, the quantitative contribution of protist-mediated HGT to the overall environmental resistome remains poorly characterized. While laboratory studies have provided direct proof-of-concept for intra-phagosomal plasmid transfer (13), the frequency, ecological significance and long-term persistence of such transfer events in natural aquatic and soil environments are unknown. Culture-independent approaches—including Hi-C chromosome conformation capture metagenomics, which physically links mobile elements to their host chromosomes in situ—could provide quantitative estimates of HGT rates within environmental *Acanthamoeba* populations without the need for cultivation. Coupling such approaches with stable isotope probing would allow the active, replicating bacterial fraction within amoebae to be distinguished from passively ingested organisms, providing a more accurate picture of genuine intracellular AMR evolution.

Beyond Hi-C metagenomics, single-cell sequencing technologies could resolve the transcriptional and genomic heterogeneity of individual bacterial cells within a mixed intracellular community, distinguishing amoeba-trained subpopulations from bystander cells that would otherwise be averaged out in bulk sequencing. Spatial transcriptomics applied to infected *Acanthamoeba* would allow gene-expression programs to be mapped directly onto their subcellular location within the phagosome, cyst wall or cytoplasm, potentially revealing spatially restricted stress responses that bulk approaches cannot detect. Long-read sequencing platforms, meanwhile, are particularly well suited to resolving the structure of large plasmids and mobile genetic elements—such as the *bla_VIM-2_*-carrying plasmid described in Section 3.3—that are difficult to assemble accurately from short-read data alone, allowing the genomic context of horizontally transferred resistance genes to be reconstructed with confidence. The clinical value of such molecular approaches is already evident outside the amoebal context: a recent review of healthcare-associated infections highlighted MALDI-TOF mass spectrometry, whole-genome sequencing and metagenomics as key advances enhancing rapid detection and control of nosocomial pathogens (49), underscoring the translational potential of applying comparable tools to protist-mediated AMR surveillance.

Second, the immunological consequences of amoebal passage require systematic *in vivo* characterization. Current evidence for adaptive immune evasion by amoeba-conditioned bacteria rests largely on ex vivo cell culture experiments and genomic inference (21). Controlled infection models comparing the virulence, antigen presentation evasion, T cell activation capacity and vaccine-induced protection against amoeba-passaged versus axenically grown bacteria of the same species are urgently needed. Such experiments would definitively establish whether environmental *Acanthamoeba* exposure constitutes a clinically meaningful ‘immune training’ event and would provide the evidence base for antigen selection in next-generation vaccines targeting amoeba-associated pathogens.

Third, the diversity of the *Acanthamoeba*-associated microbiome across different environments, host genotypes and geographic regions is incompletely catalogued. High-throughput amplicon sequencing of the 16S rRNA gene combined with shotgun metagenomics applied to environmental *Acanthamoeba* isolates from a global panel of hospital water systems, recreational water bodies and agricultural environments could reveal the full spectrum of amoeba-resisting bacteria and their resistance gene repertoires. Particular attention should be paid to resistance genes conferring resistance to last-resort antibiotics, including colistin (mcr-1 to mcr-9), carbapenems (*blaKPC*, *blaNDM*, *blaOXA-48*) and ceftazidime–avibactam combinations, as the intra-phagosomal spread of these determinants would have immediate and severe clinical consequences.

Fourth, anti-encystation strategies represent an underexplored but highly promising avenue for simultaneously disrupting both *Acanthamoeba*-mediated AMR amplification and amoebal pathogenesis. The multi-omics atlas of *A. castellanii* encystment (19) and the phosphoproteomic mapping of early encystment signals identify specific kinase cascades and cellulose biosynthesis enzymes as potential drug targets. Compounds that block encystment would not only prevent the formation of disinfection-resistant cysts but would also eliminate the protective niche that enables bacteria to survive antibiotic treatment and acquire resistance determinants. The CRISPR–Cas9 genetic tools now available for *A. castellanii* (41) provide the means to validate such targets systematically using loss-of-function screens.

Fifth, the integration of amoebal ecology into computational models of AMR dissemination represents a critical modeling frontier. Current mechanistic and epidemiological models of environmental AMR spread treat bacterial populations as freely mixing entities and do not account for the protected, intracellular reservoir represented by amoebae. Incorporating protist–bacteria population dynamics, cyst persistence curves, HGT rate parameters and disinfection efficacy data into agent-based or ordinary differential equation models would substantially improve the predictive accuracy of AMR emergence forecasts and enable evidence-based evaluation of intervention strategies—such as targeted amoebicidal treatment of hospital water systems—before costly field implementation.

## 9 Concluding remarks

*Acanthamoeba* occupies a uniquely significant position at the intersection of environmental microbiology, infectious disease, immunology and antimicrobial resistance. Its role as a microbial Trojan horse—harboring, protecting and amplifying resistant bacteria while simultaneously serving as an evolutionary training ground for intracellular pathogenesis and adaptive immune evasion—positions it as a critical but underappreciated node in the global AMR crisis. The convergent lines of evidence reviewed here collectively argue for integrating protist–bacteria interactions into AMR surveillance, water-safety management, vaccine antigen design and infection-control protocols.

A One Health approach that accounts for the role of environmental protists in resistance gene dissemination and immune evasion training is both scientifically justified and practically necessary. The remarkable conservation of phagocytic and immune evasion mechanisms between *Acanthamoeba* and human macrophages means that disrupting protist–bacterial symbioses has direct implications for preventing and treating human infections at multiple levels—from limiting resistance gene flow in the environment to designing vaccines that account for the antigenic plasticity conferred by amoebal passage. As the AMR crisis continues to intensify, the ubiquitous *Acanthamoeba* may prove to be one of its most consequential but addressable ecological and immunological drivers.
